# Determination of cut-off point of cross-sectional area of median nerve at the wrist for diagnosing carpal tunnel syndrome

**Published:** 2017-10-07

**Authors:** Majid Ghasemi, Sanaz Masoumi, Behnaz Ansari, Mahboobeh Fereidan-Esfahani, Seyed Morteza Mousavi

**Affiliations:** 1 Department of Neurology, School of Medicine, Isfahan University of Medical Sciences, Isfahan, Iran; 2 Department of Life Science, School of Sciences, University of British Colombia, Vancouver, Canada

**Keywords:** Carpal Tunnel Syndrome, Electrophysiology, Ultrasonography

## Abstract

**Background:** The most common entrapment mononeuropathy of the upper extremity is carpal tunnel syndrome (CTS). It consists 90% of entrapment neuropathies. The purpose of this study was to compare cross-sectional area (CSA) of the median nerve at the wrist in CTS patients and healthy controls and define the best cut-off point of CSA to differentiate patients and controls in Iranian population.

**Methods:** In this study, 45 patients with confirmed idiopathic CTS and 62 healthy controls were evaluated. Based on electrophysiological findings, patients were divided based on CTS severity into three groups of mild, moderate and severe. The largest CSA was measured at the level of distal wrist crease which is consistent with carpal tunnel inlet.

**Results: **Mean CSA was 0.124 ± 0.031 mm^2^, 0.146 ± 0.028 mm^2^ and 0.194 ± 0.062 mm^2^ in mild, moderate and severe CTS patients respectively, and 0.077 ± 0.011 mm^2^ in controls. Our results showed that participants with CSA > 0.010 had CTS with 100% specificity and 83.12% sensitivity.

**Conclusion:** It is possible to diagnose CTS by measuring CSA and using above-mentioned cut-off point.

## Introduction

The most frequent entrapment neuropathy is the neuropathy of median nerve at the wrist, called carpal tunnel syndrome (CTS), which occurs as a result of compression of the median nerve at the carpal tunnel. CTS is one of the leading causes of hand dysfunction. Median nerve entrapment in the carpal tunnel causes clinical symptoms such as pain, numbness, and tingling.^1^^-^^4^ The prevalence of CTS has been shown to be 5.8% and 0.6% in women and men, respectively.^5^ A few studies conducted in the United States of America showed 0.2% of all outpatient visits had been due to CTS.^6^ Although electrophysiological tests have been considered as the gold standard for CTS diagnosis and distinguishing the different severities of disease,^7^^,^^8^ their sensitivity ranges from 49% to 86% and their false negative range is between 16% and 34%.^9^^,^^10^ This variability seems to be attributed to different study methods and measurement techniques, in addition to demographic factors such as gender, age and weight.^11^

In the last few years, it has been shown that ultrasonography is a useful diagnostic tool for CTS diagnosis because of its noninvasiveness, lower cost and wide availability.^3^^,^^12^ The cross-sectional area (CSA) of the median nerve at different locations, can be measured for this aim. Different studies showed that CSAs of the median nerve at different levels of the carpal tunnel are significantly greater in CTS patients as compared to normal population. Various studies suggested different cut-off points for the diagnosis of CTS.^13^^,^^14^ In previous studies, cuto-ff point of cross-sectional area at tunnel inlet in CTS patients ranged from 6.5 to 15 mm^2^.^15^^-^^18^

The aim of this study was to compare CSA of the median nerve at the wrist in patients with CTS and normal controls and define the best cut-off point of CSA to differentiate patients and controls in Iranian population.

## Materials and Methods

This case-control study was conducted in a one-year period in the neuromuscular department of teaching hospitals of Isfahan, Iran. According to sample size estimation, at least 14 patients were needed to be enrolled in each group, but for more accurate results we enrolled 45 patients with established idiopathic CTS (within two weeks of electrophysiological examination) in 77 of their wrists and 62 healthy controls, with 124 normal wrists.

All electrodiagnostic (EDX) studies were done before ultrasonography evaluation by a neurologist with neuromuscular expertise. According to EDX results, patients were categorized as mild, moderate, and severe CTS based on the following criteria.^8^ Mild: Prolonged distal sensory nerve action potential-latency (SNAP-L) and/or median mixed nerve action potential-latency (MNAP-L), and normal distal compound muscle action potential-latency (CMAP-L), and normal amplitudes of all responses. Moderate: Prolonged SNAP-L and CMAP-L, and with or without diminished amplitudes of all tested responses. Severe: Unobtainable median sensory nerve action potential plus low-amplitude or unobtainable median compound muscle action potential and, if present, prolonged CMAP-L.

Patients with underlying diseases that may affect CSA of median nerve independent of CTS, such as wrist trauma, cervical radiculopathy, polyneuropathy, and CTS patients with previous corticosteroid injection were excluded. 

All participants filled out an informed consent before the study. All ultrasonography evaluations were done by means of a 13-MHz (SonoSite) linear array Transducer. The examiner was blinded to clinical symptoms and EDX results. Patients were asked to lie on the bed while their forearms are extended. They were rested in the supine position on a smooth surface, and their fingers were semi-extended. The largest CSA was measured at the wrist as described by Ziswiler, et al.,^19^ at the beginning of the examination by performing gray scale examination.

Data were analyzed by using SPSS software (version 18, SPSS Inc., Chicago, IL, USA), presented as the mean ± standard deviation (SD). The analysis of variance (ANOVA) was applied for comparing continuous variables. Receiver operating characteristic (ROC) curve was used to determine optimal cut-off values of the median nerve inlet CSA. In addition, we used analysis of covariance (ANCOVA) to neutralize the confounding effects of different factors on CSA. The area under the curve (AUC) was calculated. P ≤ 0.050 was statistically considered significant.

## Results

**Figure 1:**
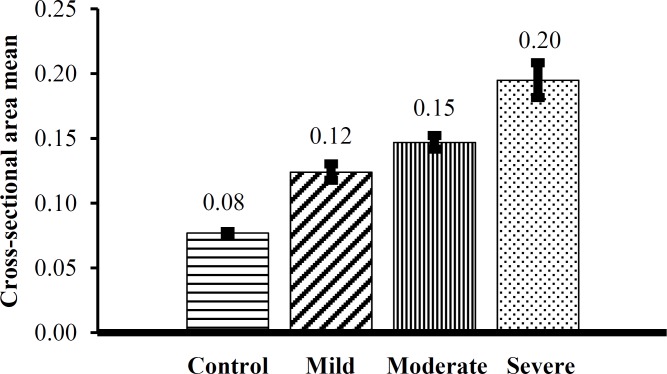
Mean cross-sectional area (CSA) of median nerve in different groups

Table 1 and figure 1 both show the descriptive statistics of the median nerve CSA at the wrist.

**Table 1:** Descriptive statistics of median nerve cross-sectional area (CSA) at the level of carpal tunnel inlet in mild, moderate, and severe carpal tunnel syndrome (CTS)

**Subjects**	**Mean ± SD**	**Range**
Control	0.077 ± 0.012	0.05-0.10
Mild	0.124 ± 0.032	0.08-0.18
Moderate	0.147 ± 0.028	0.08-0.19
Severe	0.195 ± 0.063	0.11-0.32
Total	0.106 ± 0.049	0.05-0.32

SD: Standard deviation

**Figure 2:**
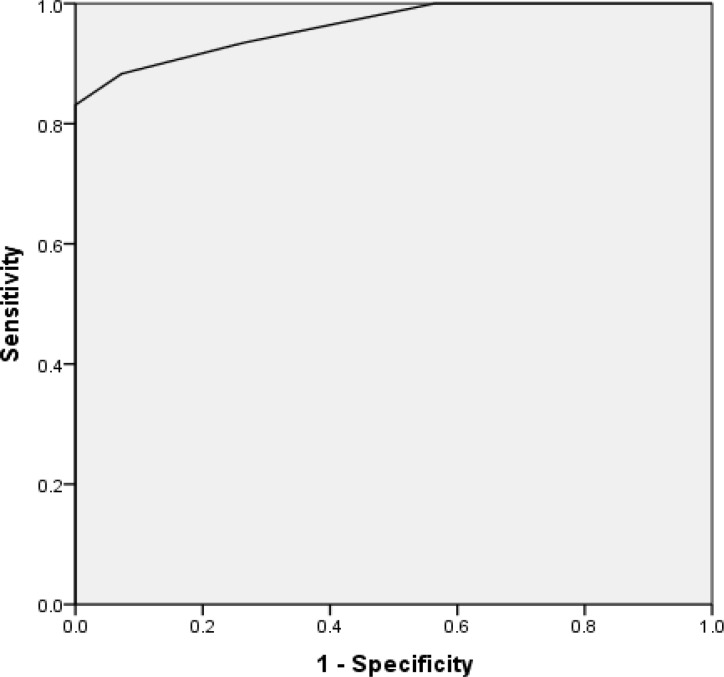
Receiver operating characteristic (ROC) curve

As the aim of our study was a differentiation between healthy controls and CTS patients using CSA, ROC curve was used to define a cut-off point for the diagnosis of CTS. Different values of CSA were considered as cut-off points. Sensitivity and specificity (percent of correct detection of controls and patients) were determined for each cut-off point (Figure 2).

As high sensitivity and specificity were very important in this study, a point of ROC curve which had the highest sensitivity and specificity was defined using Youden index, as the cut-off point. So our results showed that participants with CSA > 0.01 had CTS with 83.12% sensitivity and 100% specificity. AUC of ROC curve was calculated equal to 0.962, which is statistically significant (P < 0.001), and showed the prediction ability of CSA is not based on chance.

ANOVA showed that mean CSA-D has a statistically significant difference between controls and different groups of patients (P < 0.001). Our analyses showed that the mean age has a significant difference between case and control groups; therefore, ANCOVA was used to adjust this difference. However, there was still a significant difference of mean CSA-D between two groups with adjusting to age (P < 0.001).

## Discussion

Our results demonstrated that the largest mean value of CSA is (0.194 mm^2^) in severe CTS patients and the smallest mean value is (0.124 mm^2^) in mild CTS patients. Thus, there is the statistically significant difference between the mean CSA in severe and mild CTS patients. In addition, the results of this study showed that mean CSA of the median nerve at the wrist in CTS patients was significantly different from healthy controls and the best cut-off point of CSA for diagnosing CTS is 0.543 mm^2^, which is an appropriate value.

Mohammadi, et al. studied the diagnostic significance of median nerve CSA in severity grading of CTS. Unlike our results, they found that the difference in CSA of the median nerve in different severities of CTS was not statistically significant in either the tunnel inlet or outlet. They also concluded that ultrasonography does not have any diagnostic value for grading the severity of CTS.^16^

Similar to our study, Sarraf, et al. studied the best cut-off point for the median nerve CSA at the level of carpal tunnel Inlet. According to their results, mean CSA and perimeter in patients and healthy controls were significantly different and the best cut-off point for CSA was 10.5 mm^2^ with 80% and 76% sensitivity and specificity, respectively. Ultimately, they believed that median nerve CSA at the wrist is helpful as a diagnostic tool for CTS.^18^

Dalili, et al. concluded that the CSA of median nerve at both inlet and outlet of the carpal tunnel has a considerable association with CTS diagnosis and could be used for diagnosis of CTS, which is similar to our findings.^7^

Also, we found that the sensitivity and specificity of CSA equal to 0.105 for diagnosing CTS is 83.1%, 100%, respectively. 

In contrast to our results, Yazdchi, et al. concluded that the sensitivity and specificity of the median nerve ultrasonography for diagnosing CTS were low and ultrasonography could not replace nerve conduction study which is the gold standard of this diagnosis, but it might provide useful information.^13^

Ziswiler, et al. investigated the largest CSA of the median nerve at the wrist and found a mean value of 12.2 mm^2^ in CTS patients and 7.9 mm^2^ in controls. Moreover, a cut-off point of 10 mm^2^ showed 82% and 87% sensitivity and specificity, respectively.^19^

In Nakamichi and Tachibana study, with the median nerve CSA cut-off point value of 12 mm^2^, 67% sensitivity, 97% specificity, and 82% accuracy were reported.^20^

Ulasli, et al. studied the reasons for using swelling ratio in sonographic diagnosis of CTS and a liable method for its calculation. Their results showed that the greatest sensitivity (99%) of the median nerve CSA is where the cut-off point is considered 10 mm^2^. However, it had a low specificity value (71%), which increased the false positive rate.^21^

## Conclusion

The current study showed that diagnosis of CTS is possible by measuring CSA. According to our findings, the most excellent cut-off point of median nerve CSA at the the wrist is 0.105 (with 100% specificity and 83.1% sensitivity) and 0.095 (more sensitive than the first cut-off point). It is an appropriate method in order to diagnose CTS.

## Notes:

**How to cite this article:** Ghasemi M, Masoumi S, Ansari B, Fereidan-Esfahani M, Mousavi SM. Determination of cut-off point of cross-sectional area of median nerve at the wrist for diagnosing carpal tunnel syndrome. Iran J Neurol 2017; 16(4): 164-7.
